# A Treatise on Venereal Diseases

**Published:** 1853

**Authors:** 


### REVIEWS.

*A Treatise on Venereal Diseases*, by A. VIDAL, (de CASSIS), Surgeon to the Venereal Hospital of Paris, etc. Translated and edited by GEO. C. BLACKMAN, M. D. New York, 8vo, pp. 499.

WHAT do we know of Venereal diseases? Has the profession any positive knowledge whatever concerning their history, origin, causes, symptoms, treatment, or classification? These may seem idle and foolish questions to many of the readers of our Journal; but can anyone answer them? "Certainly," exclaims some honest, sensible, practical observer, "we know that gonorrhœa, or blennorrhagia as the books now-a-days call it, and syphilis, are the only true Venereal diseases; that they are entirely distinct in their essential natures; that the former is almost strictly local, whereas the latter is with nearly equal positiveness constitutional; that the former, compared with the latter, is quite a harmless affection; that the former is entirely curable without the use of a constitutional specific remedy, etc., etc." But stop, my good friend: we acknowledge these to be very rational and practical views; but where did you get them? Have you *experimented*?

Are you familiar with the writings of Ricord? Have you read the treatises of Maisonneuve and Montanier, Egan, Christopher, Sperino, Waller, Robert, and the volume whose title stands at the head of this article; or the recent discussions by Malgaigne and others in the French Academy; or the report of Dr. Auzias? We take it for granted that your studies have not embraced even the greater part of this enumeration, else you would speak a little less confidently, if not with less good reason; and we hope for the sake of your self-complacency and peace of mind, but more particularly for the welfare of your patients, that you will retain your present views, and not involve yourself in the turmoil and confusion now prevailing among the syphilographers of Europe.

It is a remarkable fact, that of the eight or ten books on Venereal diseases published in France and England within the past three or four years, most of them authored by men of great celebrity and acknowledged ability, hardly any two agree upon any of the essential points of doctrine or practice. And it is still more worthy of notice, that the majority of these very recent works are opposed to the common-sense views held by the great mass of the profession the world over, and so fully substantiated, in our humble judgment, by the observations and experiments of the renowned Ricord. That this acute observer and able expounder of Venereal truths has allowed his zeal and self-confidence to carry him somewhat beyond the bounds of prudence, and has made himself a little obnoxious to his brethren in Paris and London by prematurely boasting of achievements which subsequent observations have not entirely perfected, cannot be denied; but that the majority of the main principles for which he contends are true, the experience of the profession, in this country at least, is almost unanimously agreed. Whatever his opponents may say of him, however, to him "is undoubtedly due the credit of having introduced a new method of investigating the nature of Venereal diseases—namely, that of innoculating the products of the different affections which arise, with the point of a lancet on some apparently healthy part of the skin of the affected person." And it may be as well to state here, by way of establishing a point of departure in the discussion of this subject, the great fundamental 'propositions' or conclusions at which he has arrived by this method of investigation, and which have been adopted also by M M. Maisonneuve and Montanier.

"1.—That blennorrhagia and chancre are two entirely distinct affections.

2.—That blennorrhagia is an inflammatory contagious affection.

3.—That a chancre always produces a specific virus.

4.—That blennorrhagia never gives rise to constitutional syphilis.

5.—That a chancre always produces a constitutional disease when it becomes indurated.

6.—That a non-indurated chancre is always a local disease, and is never accompanied or followed by secondary affections.

7.—That a chancre is the only origin of syphilis.

8.—That pus derived from a primary affection (i. e. from a chancre or suppurating bubo) is the only contagious element in the disease.

9.—That no secondary or tertiary disease is capable of being communicated by contagion.

10.—That blennorrhagia may arise spontaneously and never produces any disease but blennorrhagia.

11.—That a chancre is always of necessity the result of a chancre.

12.—That there is but one syphilitic virus." *

That M. Ricord has succeeded in establishing the greater number of these propositions, we think no one can doubt who will take the trouble to examine the evidence presented in his notes to 'Hunter on Venereal,' his work on 'Innoculatino,' and his more recent 'Letters on Syphilis.' And not only so, but, as just stated, there is a remarkable correspondence between the most of these conclusions and the experience of surgical practitioners in general throughout this country, and, as far as we know, in France and England also. Indeed, the only way in which we can explain the bitter opposition made to them by so many lecturers and writers on the other side of the Atlantic, is by a reference to personal feelings of envy and jealousy. That M. Vidal, who has heretofore enrolled his name among the most distinguished surgical writers of this or any other age by his truly admirable work, entitled 'Pathologie Externe,' should have permitted himself to become embroiled in this discussion, is particularly unfortunate; for the violent partizan feelings exhibited in the work before us can add nothing to the stability of his wide-spread reputation, but will seriously detract from the confidence the profession have heretofore placed in his teachings. The book strikes us indeed, as an elaborate and systematic attack upon M. Ricord, notwithstanding the general assertions to the contrary contained in the first paragraph of the preface. Hav-

*British and Foreign Review.

ing placed himself therefore in the front rank of the opposition, M. Vidal seems determined to give no quarter, and boldly defies his opponent upon every one of the aforementioned propositions—not however 'with the weapons of his (M. Ricord's) choice,' but by a glittering display of names and a flourish of rhetoric that is anything but satisfactory to the sincere seeker after truth. The great array of authority brought forward by M. Vidal may serve very well as a corps of reserve in case of defeat, but having aspired to the place of a champion—the Goliath of his regiment—it is a little remarkable that he should have declined a single-handed combat. But such is undoubtedly true, for after a careful examination of his work, we find that by very far the great majority of his asserted facts are the results of observations and experiments, in which he not only did not take part, but of which he was not even a witness.

We would hot have our readers suppose, however, that we place a very low estimate upon the labors of our author, for as a collection and presentation of authority in favor of the position he has taken, his work has probably no equal. It is only in the light of an annihilator of M. Ricord, that our judgment pronounces it a failure. The latter gentleman, notwithstanding the undignified braggadocio manner with which he urges his claims, and notwithstanding the loss of a few of his numerous laurels, still stands before the profession as the great Venereal apostle and the zealous devoted student of nature.

The work of M. Vidal opens with a copious *Introduction*, expressive of the author's views of Venereal diseases in general and his opposition to M. Ricord in particular.—The body of the work is divided into four parts: 1st, an elaborate exposition of the whole subject of *Primitive Ve-*

*nereal Diseases*, including under this head gonorrhœa and its local effects (stricture, orchitis, cystitis, &c.,) chancre, and bubo : 2nd., *Consecutive* or *Constitutional Venereal Disease*, embracing syphilitic eruptions, iritis, falling of the hair, affections of the mucous membrane, ostitis, penostitis, &c., or, in short, all those effects denominated by Ricord secondary and tertiary : 3rd, an instructive discussion of the subject of *Infantile Syphilis*, including the question of its hereditary descent, and its transmissibility from child to nurse and nurse to child : 4th, the *Prophylaxis* of *Venereal Diseases.*

Looking at the work as a counter-blast to M. Ricord, we shall attempt in the remainder of this article to give a brief synopsis of some of the arguments and facts produced by the author in justification of his opposition.

In combating M. Ricord's first proposition, namely : 'that blennorrhagia and chancre are two entirely distinct affections,' M. Vidal asserts :

1st.—'That gonnorrheal matter is capable of producing, chancre.' The truth, however, of this statement he bases as far as we have been able to ascertain, solely upon the authority of Hunter, and a single successful experiment said to have been made by a man named Andre. Now without attempting any explanation of the probable error committed by Hunter, farther than to say that he was almost ignorant of the existence of chancres within the urethra, and without calling in question the authority of M. Andre, we must confess that it strikes us as remarkably singular that M. Vidal, who has had an experience of ten years at the *Hospital du Midi*, and who, it is presumed, prescribes for scores of Venereal patients every day of his life, should have been willing to leave the proof of so important a point to anyone else whatsoever, when he could so easily decide it for himself. Indeed we can hardly believe that he has not performed the experient of inoculation with gonorrhœal pus over and over again. If our suspicions are true, his want of success is as evident as his want of candor. But to take a more charitable view of the subject, by supposing that M. Vidal's *morality* has deterred him from inoculating his patients with what he considered a virulent, poisonous matter, we would ask if the profession is really any wiser for his expression of opinion. The experiments of Hunter were made half a century ago, but notwithstanding the general confidence the professional world to this day place in his authority, we venture to say that there are not ten surgeons either on this or the other side of the Atlantic who believe with him in the inoculability of gonorrhœal matter, and if M. Vidal wishes to convert the world to this doctrine he must present us with no less evidence than successful experiments performed by his own hands and in the presence of not a few responsible witnesses.

2nd.—'That the matter of a mucous balano-posthitis (simple balanitis) may be successfully inoculated, and that too when there is no ulceration nor the slightest erosion or solution of continuity in the mucous membrane.' Now here is a case about which the reader will no doubt think there can be no mistake. There is here no urethral chancre for M. Ricord and his followers to fall back upon, and the existence of an ulcer upon the glans penis or prepuce is too easily ascertained to admit of any error in diagnosis. But has M. Vidal demonstrated that the matter of balanitis will produce chancre? Hear what he says: 'I have not repeated these experiments (referring to some experiments of M. Bartholi upon the same point)

because *the syphilitic nature of balanitis under the form which is called simple catarrhal, I have never doubted,* since I have been able to watch for a length of time the patients in whom it occurs.'

3rd.—'That both simple uncomplicated gonorrhœa and balanitis may give rise to suppurating buboes, the pus of which is inoculable.' But upon this point the author does not present any evidence of his own or any detailed observations of others.

4th.—'That gonorrhœa and balanitis are often followed by constitutional symptoms, such as eruptions, falling of the hair, &c.'—effects that are referred by Mr. Ricord and others only to pre-existing chancre. This is one of the author's strong points, and so convinced is he of its truth, and of the stupidity or obstinacy of those who do not believe with him, that he hardly condescends to argue the question, but contents himself with a bare reference to the authority of others and with a frequent repetition of what he says is a well-known fact. The following extract will give the reader an idea of the manner in which this branch of the subject is treated.

'But admit for a moment the existence of chancre deep in the urethra, provided that it is rare and exceptional, and that we have the candor to acknowledge. Now compare the frequency of urethral chancres with the number of consecutive syphilitic accidents which have been seen to follow urethral discharges, and the *enormous number of the latter* compared with the former cases, will be at one apparent. Physicians who devote special attention to diseases of the skin, as, for example, M M. Cazenave, Martins, and Legendre, will tell you that *blennorrhagia produces as many cutaneous affections as chancre.*' * Now deduct some cases

*The Italicisms are our own.

of blennorrhagia which you may carry to the column of urethral chancres (if you admit this existence), there will remain a *considerable number* of eruptions which can be attributed only to blennorrhagia. This truth has been pro claimed before the whole academy of medicine, particularly by men who have long been observers, and who have been committed to no particular theory in syphilography, such as M M. Moreau, J. Cloquet, Velpeau, P. Dubois, &c.

'Now, admit still further, that every specific syphilitic discharge is but a symptom of chancre. There will still, however, always remain a form of inflammation of the mucous membrane, a sero-purulent discharge—in fine, a blennorrhagia, which by its progress, its duration, its accidents, its complications, can never be referred to an inflammation like that produced by simple irritants, by foreign substances, or by ammoniacal injections, such as that which Swediaur produced upon himself. These simple inflammations are not communicated with the same characters from one individual to another; they are not followed by the accidents of metastasis; they do not continue a year as did the blennorrhagia which that student gave himself, by applying between the prepuce and glans, pledgets dipped in gonorrhœal matter.* All these effects, which are not denied by my opponents, proceed from a single cause, which does not simply irritate the mucous membrane, but which penetrates the system. Now this cause we call a vice or virus; he may say that it is not syphilitic, that it is gonorrhœal. We come then to the conclusion of Benj. Bell, and admit a double virus, or, more properly speaking, we apply two names to the same virus, which really requires much less expense of the imagination, much less

* B. Bell, on Venereal.

research, and much less time since it is already made. M. Beaumes also admits a double virus, that of chancre and that of blennorrhagia; the action of the latter, in his opinion, is not confined to that of irritation, it is absorbed, and then it does terminate in the production of primitive accidents, such as bubo, opthalmia, arthritis, but it gives rise to such symptoms as are described in the following remarkable passage: 'I can affirm,' said M. Beaumes, 'that during the past three years alone, I have seen at the *Hospice de Antiquaille,* exclusive of my practice in the city, five cases of *simple blennorrhagia,* where I was certain that there was no chancre in the urethra, which cases were followed after some time, by constitutional symptoms, such as *well-marked rounded ulcers on the tonsils; mucous tubercles at the commissures of the lips, about the arms, and on the scrotum; syphilitic ecthyma, furfuraceous squamous, and papular eruptions, &c., &c.* In two of these cases, on the seventh or eighth day from the commencement of the blennorrhagia, I inoculated with muco-pus by three punctures on each thigh, without any result.' M. Beaumes then admits, that urethral discharges may be produced: 1st, by a chancre; 2nd, by a virus which is not chancrous, but which gives rise to the accidents already mentioned; 3rd, by an irritation or inflammation, which produces but sympathetic effects. M. Ricord is of the opinion that M. Beaumes has arrived at these conclusions from conciliatory motives. For my own part I believe that M. Beaumes has, by a long and directly opposite method to M. Ricord, formed the opinion that there are urethral discharges without any chancre, which are virulent and capable of giving rise to consecutive syphilitic accidents; only, these accidents may be less frequent, less profound.'

The above extract contains the only approach to any positive evidence bearing upon this important question that we have been able to find in the whole work. In fact, as before stated, M. Vidal seems to take it for granted that nobody will deny that gonorrhœa and balanitis are frequently followed by secondary and tertiary accidents, and continually refers to it as an undisputed fact. Now with all due deference to the author and the American editor and the numerous authorities cited by both, we put the question to those of our readers who are familiar with Venereal diseases; and we are disposed to believe that there are not a few of them who possess no little practical knowledge on this branch of surgery: how many cases of simple gonorrhœa have you known to be followed by consecutive effects such as cutaneous eruptions, falling of the hair, ulcers of the tonsils, iritis, &c? For ourselves, notwithstanding we have made this subject something of a speciality, both in private and hospital practice, for several years past, we must confess that we have yet to meet with the first case of this sort. Indeed our practice would not even justify us in admitting with Acton (who the American editor seems to think out-Ricords Ricord himself,) that 'in gonorrhœa the system is so modified as to become affected by rheumatism.' But supposing we do allow that gonorrhœa treated in the ordinary way by copaiba, cubebs, &c., is sometimes followed by cutaneous symptoms, does this prove that the system is under the influence of a specific virus? Who does not know that the use of copaiba for other diseases as well as Venereal, is often followed by a species of rash or urticaria? and who does not know that the slightest gastric derangement produced by any cause whatever will in many persons give rise to affections of the same nature? We do not pretend to say that all the cases of cutaneous eruptions observed by M M. Vidal, Beaumes, and others are referable to causes of this character, but we do say that the few cases of reputed constitutional infection from gonorrhœa prove nothing whatever upon this point, when compared with the thousands upon thousands of cases in which no such effects have been observed.

The last assertion of M. Vidal that we shall touch upon concerning this question is, that the matter of chancre is as often the cause of gonorrhœa as that of gonorrhœa itself. Again, we must ask does M. Vidal present any facts to prove this? Has he introduced the matter from chancre into the urethra of any of his patients or students?—This it seems to us would be a very harmless experiment, if the doctrines of M. Vidal are true; for a patient laboring under chancre or bubo could not be hurt much worse by a gonorrhoea, and even with the probability of complicating the case a little, we doubt not that M. Vidal's zeal to overthrow the conclusions of M. Ricord has at least suggested to him the performance of such an experiment, which no doubt would have been performed, had be been willing, as he avers, to contend with M. Ricord with the weapons of the latter surgeon's choice. But no such experiment is reported, and the reader must be content with the citation of the opinions of Hunter and others, and the author's oft-repeated assertion, that 'virulent pus, that which is the medium of the syphilitic virus, is, in my opinion the most frequent and powerful cause of blennorrhagia.'

The second of M. Ricord's propositions, *i. e.* 'that blennorrhagia is an inflammatory contagious affection,' is necessarily involved in the discussion of the first. If M. Vidal has succeeded in proving the identity of the gonorrhœal and syphilitic virus, this settles the point at once: but if he has failed to do this, it does not necessarily follow that M. Ricord is right, for it is still an unsettled question, among those who believe in the separate nature of the two diseases, whether genuine gonorrhœa always originates from a specific virus. M. Ricord and many other very distinguished teachers and writers, on both sides of the Atlantic, besides a very large number of observing practitioners not known to fame, take the negative ground, whereas an equally large and respectable proportion, the distinguished Professor of Surgery in the Louisville University among the number, have expressed themselves in the affirmative.

The third proposition of M. Ricord, *i. e.*, 'that a chancre always produces a specific virus,' seems to have been taken by M. Vidal in its largest signification and, of course, in this view, meets with an unqualified denial. But a reference to M. Ricord's work on Inoculatino proves that M. Vidal has not paid sufficient regard to the express distinction here made between a chancre when it is in a state of progressive ulceration and when undergoing suppuration. Taken in this limited sense, which M. Ricord repeatedlyi nsists upon, we cannot suppose that M. Vidal would attempt to controvert its truth.

Connected with this question are two others of considerable interest both in a scientific and, one of them at least, in a piactical point of view—we refer to the inoculation of inferior animals and the syphilization of the human subject. In regard to the former, MM. Ricord, Hunter, Cullerier and others have uniformly obtained only negative results. But does M. Vidal agree with these gentlemen?

Of course not. Indeed, he expresses his decided belief that M. Cullerier deceived himself and did succeed in some of his experiments upon dogs, rabbits, &c., notwithstanding this 'honest man and sage practitioner,' as M. Vidal styles him in his preface, asserts positively to the contrary. In farther justification of his position, M. Vidal cites the reputedly successful experiments of M. M. Diday and Auzias. But is it not a little strange that M. Vidal himself has not experimented upon this point? Our private opinion is, as stated in reference to the inoculation of gonorrhœal matter, that he has, but being unsuccessful, he had not the candor to acknowledge it.

In reference to the subject of 'syphilization,'* we again find our two champions at war. 'I wait,' says M. Ricord, 'that they may show me an individual syphilized and insusceptible of the action of the virus, who will go before the *clinique* of the *Hospital du Midi*, and defy me in camp with the weapons of my choice?'

M. Vidal reports (employing the language of M. Malgaigne before the Academy of Medicine), that 'this challenge was repeated on the 12th of August by the *Union Medicale*. On the 22nd of August M. Auzias accepted it: on the 23d of September, M. Ricord declared that he was waiting. The editor of the *Union Medicale* exclaimed: *Facts, facts!* give us rather than theories! and then gentlemen, then, the 4th of November, M. Ricord pro-

*It may be necessary to state for the benefit of those of our readers who are not familiar with all the technicalities of modern syphilography, that by the term 'syphilization' is meant the saturation of the system with the syphilitic influence by means of inoculation, so as to render the individual proof against any farther infection. The word was coined, we believe, by M. Auzias, who is not the originator, but a zealous advocate of the doctrine that it involves.

claimed that the experiments had been commenced; and the results would be communicated to the journal.—Search it well and this communication cannot be found.—Eight days afterward M. Ricord presented M. Laval* to the Surgical Society and on the 18th of November to the Academy of Medicine. But not one word about the experiments. On the 20th, M. Latour gave the case of M. Laval as the only public and authentic experiment then known. As if to break this silence, on the 9th of December M. Marechal wrote to the *Gazette des Hopiteaux* that M. Laval had presented himself to M. Ricord, that the latter had made on him, at two different localities, seven punctures, and had inserted three kinds of pus, of undoubted virulence, without any results. M. Ricord made no reply; the *Union Medicale* lisped not a word. You are aware that the report of the committee maintained the same silence. For myself, gentlemen, this silence amounts to a defeat, and yet I should have preferred a frank and public acknowledgment. . . . . . .

Here, then, is a truth of capital importance elicited by this discussion. You have read the excellent treatise of M. Ricord on syphilitic inoculation; you have seen the valuable results which he has obtained in diagnosis, prognosis, and therapeutics; you will recal to mind his nice deductions in legal medicine; all these are shaken, all totter when chancre is no longer inoculable; diagnosis becomes uncertain, prognosis deceptive, and treatment doubtful; above all, can legal medicine, which requires absolute certainty, longer dare to trust, as heretofore, to inoculation? These remarks apply directly to primary

* The great 'Venereal martyr.'

syphilis only ; there is reason to apprehend that the system is soon to suffer another check as regards constitutional syphilis.'

'For my part,' concludes M. Malgaigne, 'I regard the fact that certain persons may acquire an immunity against syphilitic inoculation as incontestably demo nstrated. If M. Ricord doubts it, I will engage to produce a young man who believes himself syphilized, and who will defy M. Ricord to produce in him a single atom of pus capable of being again inoculated. M. Ricord shall take his precautions. If he does not succeed he shall begin again: my patient declares himself ready to allow twelve hundred inoculations to be made upon him—and more, should this not be deemed sufficient.'

M. Vidal, however, does not go quite so far as M. Malgaigne, as may be inferred from the following extract.

'Now, a fact, which, in my opinion, is unquestionable, is the syphilization of a certain sphere ; in fine, a local syphilization. Thus I have seen a very active chancre on the summit of the glans spread the pus, which it secreted, over the rest of the organ, bathe the whole prepuce which was much elongated, and yet these parts were not inoculated. This same pus imbued the penis and the scrotum, which resisted its action. But one day there appeared on the superior and internal part of the thigh, a well marked chancre, which furnished an inoculable pus. It was produced by the end of the penis, which several times rested in contact with this part of the thigh. Thus, the same pus, which on the genital organs was inert, had inoculated the thigh. The genital sphere was here syphilized, or, in other words, it was under the influence of a vital reaction, which resisted the extension of the ulceration on the summit of the glans, as well as the production of other chancres. This kind of local syphilization must in all cases be admitted, otherwise every chancre would increase indefinitely, it would know no limits. The more decided this local syphilization, the more limited the chancre: phagedenic ulceration shows the absence in a certain sphere of this syphilization But let it not be supposed, on this account, that syphilization is permanent. These same parts, this same sphere, which in the patient above mentioned could not be inoculated by the pus of the chancre on the summit of the glans, became so a month afterward from an impure coitus.'

We leave the reader to judge of both MM. Malgaigne and Vidal. The former declares all of the results of M. Ricord's numerous experiments to be unscientific and truth-less, simply because one person has been found whose sys-tem resisted syphilitic inoculation; while the latter, hardly barefaced enough to attempt to establish his opposition upon so narrow a basis, tries to shuffle out of the difficulty by suggesting a ridiculous modification having no relevancy whatever to the question in dispute. We wouid call attention, however, to the closing passage of M. Vidal's chapter on this subject, wherein, having confessed that 'the facts yet produced by this prophylactic or curative syphilization are far from entitling it to a place in medical science,' he pleads in justification that they *may be of real value in assisting to destroy a system resting on a false basis.* This is the impartial investigator who, in his preface, modestly condemns the innumerable works on Venereal disease which have 'been written for the purpose, more or less candidly admitted, of establishing or *overthrowing* some particular doctrine:' who 'proposes to collect such facts and opinions as are of practical application, and which have survived the wreck of systems; and who humbly, but with an air of self-complacency closes his preface with the following paragraph.

'Lately, some syphilographers whose tenets have been shaken, have allowed themselves to descant bitterly, or in a jesting tone. They have even written in the same style. Having no motive for sharing in the sentiments which have inspired a literature of this kind, having, besides, remarked that it has neither thrown light upon, nor advanced the questions in dispute, I have abstained from it, and have endeavored to speak as clearly as possible the language of science, since it is the latter only which I have in view, and the interests of those who would become her earnest votaries.'

Space will not permit us to take up all the remaining propositions of M. Ricord, and present the arguments and facts produced by M. Vidal to prove their falsity. We shall content ourselves for the present with a few extracts from M. Vidal's article on the transmissibility of secondary accidents, which, for force of argument—argument based upon something apparently more reliable than doubtful authorities or mere opinions—and clearness of diction, is unequalled by any other in the work.

After stating the importance of this branch of the subject in a private hygienic and legal point of view, the author refers to the absurd notion held by the old writers on syphilis, who supposed that the disease could be communicated by the atmosphere, the breath, perspiration, and, in fine, by everything which surrounds and by all that is within man. He then points to the views upon the opposite extreme held by Hunter and Ricord, and attempts, with a considerable show of success, to demonstrate that truth lies between these two remote doctrines. In stating his proofs, he commences by pointing to what he says is a well-established fact, namely, that the child is often infected in the womb of the mother, (when the latter is the subject of constitutional syphilis,) and is, when born, capable of communicating the disease to its nurse by the ulcers that exist upon its mouth and elsewhere. Arguing analogically from this fact, he says, ‘if even after the cicatrization of a chancre, the system may still contain an infectious principle capable of being transmitted to another being, by multiplying the relations, diversifying the experiments, and employing different humors, we may expect to prove the transmissibility of syphilitic accidents independent of chancre or its products. Observation, here confirming analogy, should have anticipated the results of experiment, and, doubtless, in any other epoch than the present would have rendered it unnecessary, for the instances of the infection of the nurse by the child, which is seldom effected by other than secondary accidents, are exceedingly numerous, and MM. Bouchacourt and Bardinet have recently reported cases with details so precise as to leave no room for doubt.’

In regard to the infection of the nurse by the child we are almost entirely without the means of observation in this country, where mothers almost invariably suckle their own offspring; and we have therefore to rely almost entirely upon the authority of our brethren of Paris, where the miserably inhuman practice prevails among a very large class of persons of putting their infants out to be nursed, and, in many instances among the higher orders of sending them to the provinces, not only to be nursed but to be raised until they are six, ten, or twelve years of age.

Believing as we do, in the contagiousness of only the primary effects of syphilis, the explanation of these cases of the inoculation of nurses rests upon the supposition that the child contracts chancre in passing the vagina and vulva of the mother.

M. Vidal continues: 'Again, the transmissibility of the mucous tubercle, by means of intimate relations and contacts, is generally admitted. On this subject the works of M M. Lagneau, Beaumes, Cazenave, Gilbert, and of other unprejudiced observers may be consulted. In another place I shall appeal to M. Ricord himself, we shall find that he admits the infection of the nurse by the child, and the contagiousness of the mucous tubercle in the adult; but, as he expresses himself in his *Treatise*, 'it is by some incomprehensible vital process.' He admits contagion only, that is physiological inoculation, and still denies the fact of experimental inoculation.'

It may be true, as M. Vidal states, that M. Ricord admits the transmissibility of mucous tubercle, and the infection of the nurse by a child laboring under congenital secondary syphilis, but we must confess our inability to find it so in any of his published works. On the contrary, we find in his *Treatise* the following conclusions in regard to this matter.

1.—That mucous tubercle never inoculates;

2.—That it ought to be placed with the secondary symptoms, and is a proof of constitutional syphilis;

3.—That the secretion which it may produce, acting like irritating matter, causes inflammation of the tissues with which it comes in contact;

4.—That where syphilis has been transmitted to an individual from mucous tubercle, there must have been other specifically contagious symptoms at the time of the infection;

5.—That, like the other secondary symptoms, the true mucous tubercle can be transmitted only by inheritance.

Again, with regard to the transmission of the disease from the mother to child, and from the child to the nurse, M. Ricord says in his *Letters:* 'I have seen nurses, and the children they were suckling, diseased, and both parties accused of infecting the other. Generally, I have been able to trace the origin of the disease to a primary sore in one of the parties. When this has not been the case, I was called too late; five or six months, perhaps, after the child had been with its nurse. I have for many years had a number of nurses at the *Hopital du Midi*, and I had often given them children to suckle, which were sent to me from the *Maternite*, with secondary affections. Never, as far as my observation extends, were these nurses infected. On the other hand, nurses, palpably affected with secondary disease, have been able to suckle infants which were sent as being affected with syphilis, but who had had only simple eruptions of eczema, impetigo, or porrigo, and never were these children, whilst under my inspection, infected.'

In confirmation of the doctrine of the transmissibility of constitutional syphilitic accidents, M. Vidal contends that the secretion from secondary ulcers under certain circumstances can be artificially inoculated, and refers to the successful experiments of Waller, (of Prague), Puche, Cazenave, Richet, and himself. He says: 'I have experimented, and for the first time in France, have succeeded in inoculating the pustule of secondary ecthyma. At a later period, a German physician was presented to the Academy of Medicine, who had inoculated himself with a consecutive affection; this gave rise to another discussion, the result of which, in the estimation of every unprejudiced person, must have been a condemnation of the doctrines I oppose.'

The experiments of Waller, two in number, consisted in extracting blood by a cupping glass from a person laboring under secondary syphilis, and inoculating it by means of scarifications made upon individuals who had never had syphilis. In the only one of the above experiments detailed by M. Vidal, the inoculation was for some time thought to be a failure, but on the thirty-fourth day two small tubercles appeared at the point of inoculation, which in the course of fifteen days became converted into an ulcer, having the diameter of a pigeon egg, covered with a brown crust, and surrounded by a coppery red areola. Sixty-five days after the inoculation and thirty-two from the appearance of the first tubercles, an exanthematous eruption was observed upon the lower part of the abdomen, on the back, chest, and thighs, which was pronounced to be a well-marked syphilitic roseola.

The experiment of M. Vidal consisted in taking pus from a patient affected with pustular syphilis, and inserting it by means of a lancet into the skin of another, but apparently healthy part, of the skin of the same individual, and also into the skin of a young man, a student of pharmacy, who had never had Venereal disease. In the former case, pustules, similar to those from which the matter was taken, were produced in the course of a few days. In the latter, that of the student, a papule surrounded by a distinct inflammatory areola appeared on the third day, succeeded by a pustule which subsided and cicatrized, without any treatment about the fifteenth day, but reappeared on the thirty-fifth day and did not heal again under seven or eight weeks. The student, not thinking the experiment conclusive, refused to submit to treatment, but awaited the development of symptoms of general infection, which, sure enough, made their appearance about three months after the last cicatrization.

The experiments of MM. Puche, Cazenave, and Richet consisted in inoculating other parts of infected persons with pus taken from their own sores, as in the first mentioned experimnt of M. Vidal.

In still farther support of the doctrine of the transmissibility of constitutional syphilis, otherwise than by inheritance, the American editor cites the authority of Sir Astley Cooper, Mr. Liston, Mr. Hey, Sir B. Brodie, Mr. Lawrence, and a great many others, but adds no experiments or positive observations of theirs or his own.

On taking leave of M. Vidal for the present, which we do with no little regret, notwithstanding our objections to his doctrines, but more particularly to the spirit of his work, we beg leave to thank the American editor and translator for two things: first, for introducing M. Vidal to the profession of this country in a plain, but handsome, well-fitting English dress; and second, for not having himself written a book on Venereal diseases. His notes add very materially to the value of the work before us—indeed without them it would not be complete—but holding, as he does, views more ultra than even those of M. Vidal, and enjoying, as he does, such a deservedly high position among the surgeons of this country, we are glad, for our own sakes, that a foreigner stands between him and us.

